# Supplementary figures and images for: Biodynamics: A novel quasi-first principles theory on the fundamental mechanisms of cellular function/dysfunction and the pharmacological modulation thereof
Source: PLoS One. 2018 Nov 1;13(11):e0202376. doi: 10.1371/journal.pone.0202376 (PMC6211658; doi:10.1371/journal.pone.0202376)

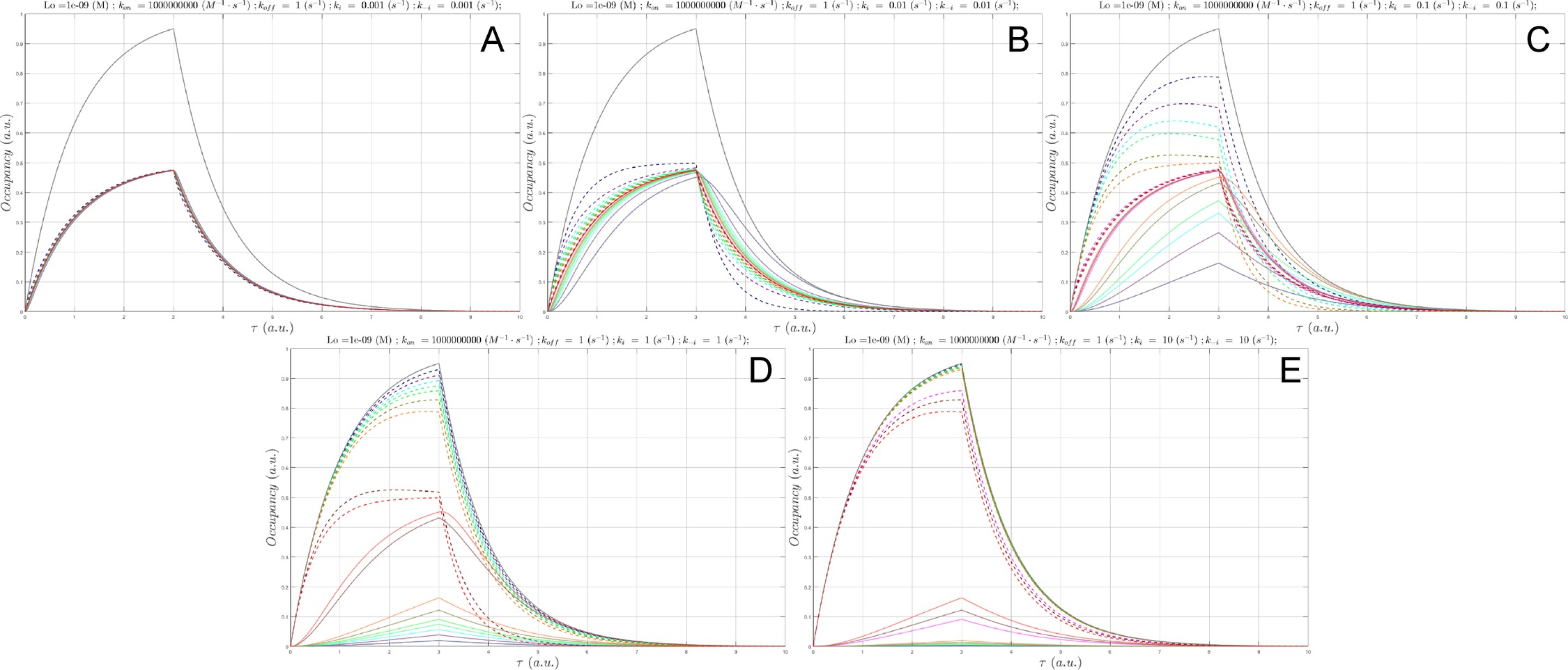

Supplement: S1 Fig — (a) Plot of Btotal(τ) (gray), c(τ) at a fixed L0 = Kd =1 nM and Λ =50 min (ki = k−i = 1x10-3 s-1, konSS = 1x10^7^ M-1 s-1), in which kon was sampled between 1x10^7^ M-1 s-1 to 1x10^9^ M-1 s-1 (solid lines color-coded from blue to red according to increasing kon), and Bfree(τ) (dotted lines color coded the same as c(τ)). (b) Same as (a), except Λ = 5 min (ki = k−i = 1x10-2 s-1, konSS = 1x10^8^ M-1 s-1). (c) Same as (a), except Λ = 30 s (ki = k−i = 1x10-1 s-1, konSS = 1x10^9^ M-1 s-1). (d) Same as (a), except Λ = 3 s (ki = k−i = 1x100 s-1, konSS = 1x10^10^ M-1 s-1). (e) Same as (a), except Λ = 300 ms (ki = k−i = 1x101 s-1, konSS = 1x10^11^ M-1 s-1). kon = 1x10^9^ M-1 s-1, the fastest kon sampled at this ki (which is 100-fold < konSS), results in nearly zero occupancy. (TIF) [file pone.0202376.s001.tif]

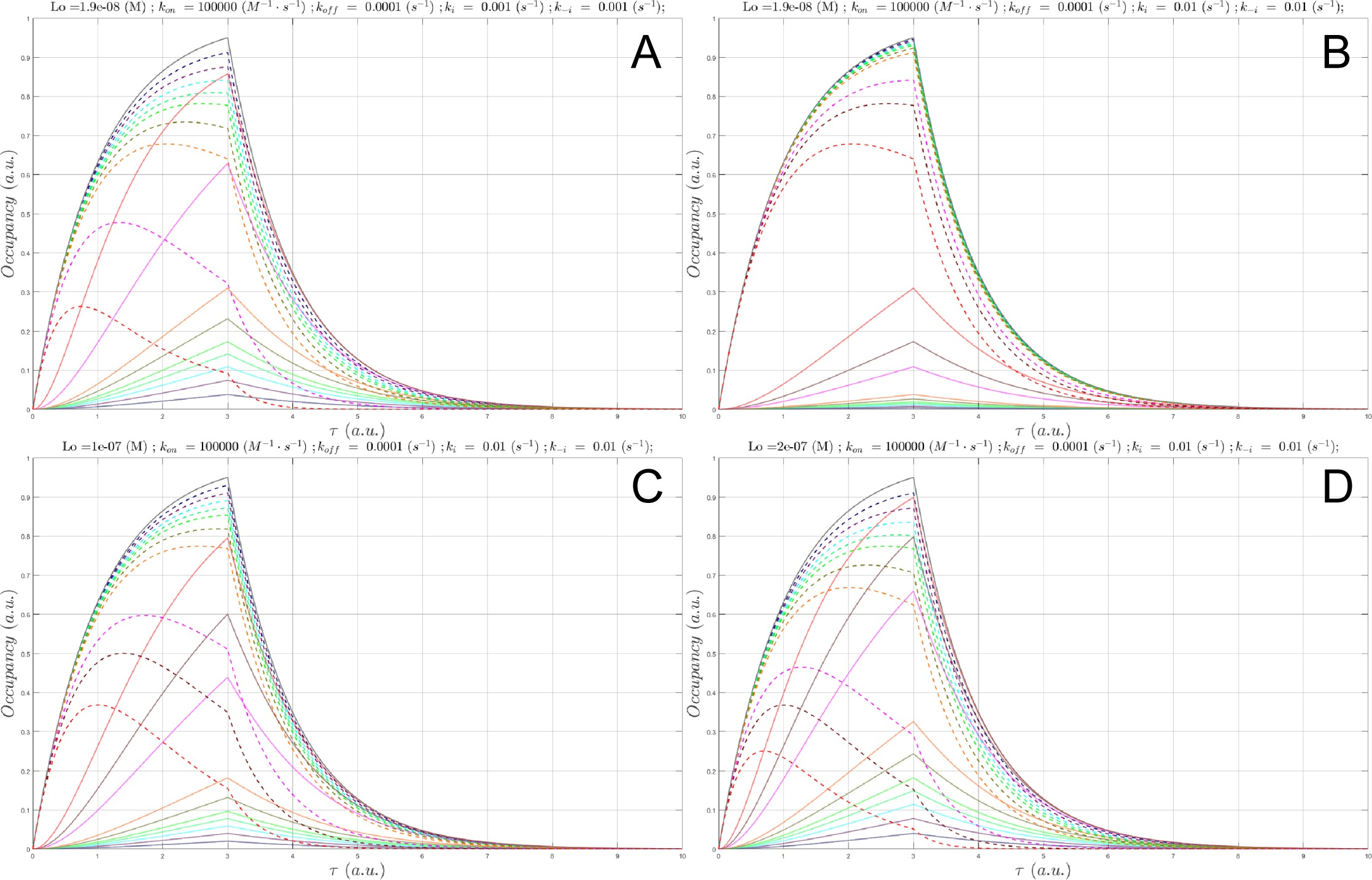

Supplement: S2 Fig — (a) Plot of Btotal(τ) (gray), c(τ) at a fixed L0 = 19 ⋅ Kd = 19 nM and Λ = 50 min (ki = k−i = 1x10-3 s-1), in which kon was sampled between 1x10^3^ to 1x10^5^ M-1 s-1 (solid lines color-coded from blue to red according to increasing kon), and Bfree(τ) (dotted lines color coded the same as c(τ)). (b) Same as (a), except Λ = 5 min (ki = k−i = 1x10-2 s-1, konSS = 1x10^8^ M-1 s-1). (c) Same as (a), except Λ = 5 min (ki = k−i = 1x10-2 s-1) and L0 = 100 nM. kon = 1x10^5^ M-1 s-1, the fastest kon sampled at this ki (which is 1,000-fold < konSS), fails to achieve the SSO profile. (d) Same as (c), except L0 = 200 nM. (TIF) [file pone.0202376.s002.tif]

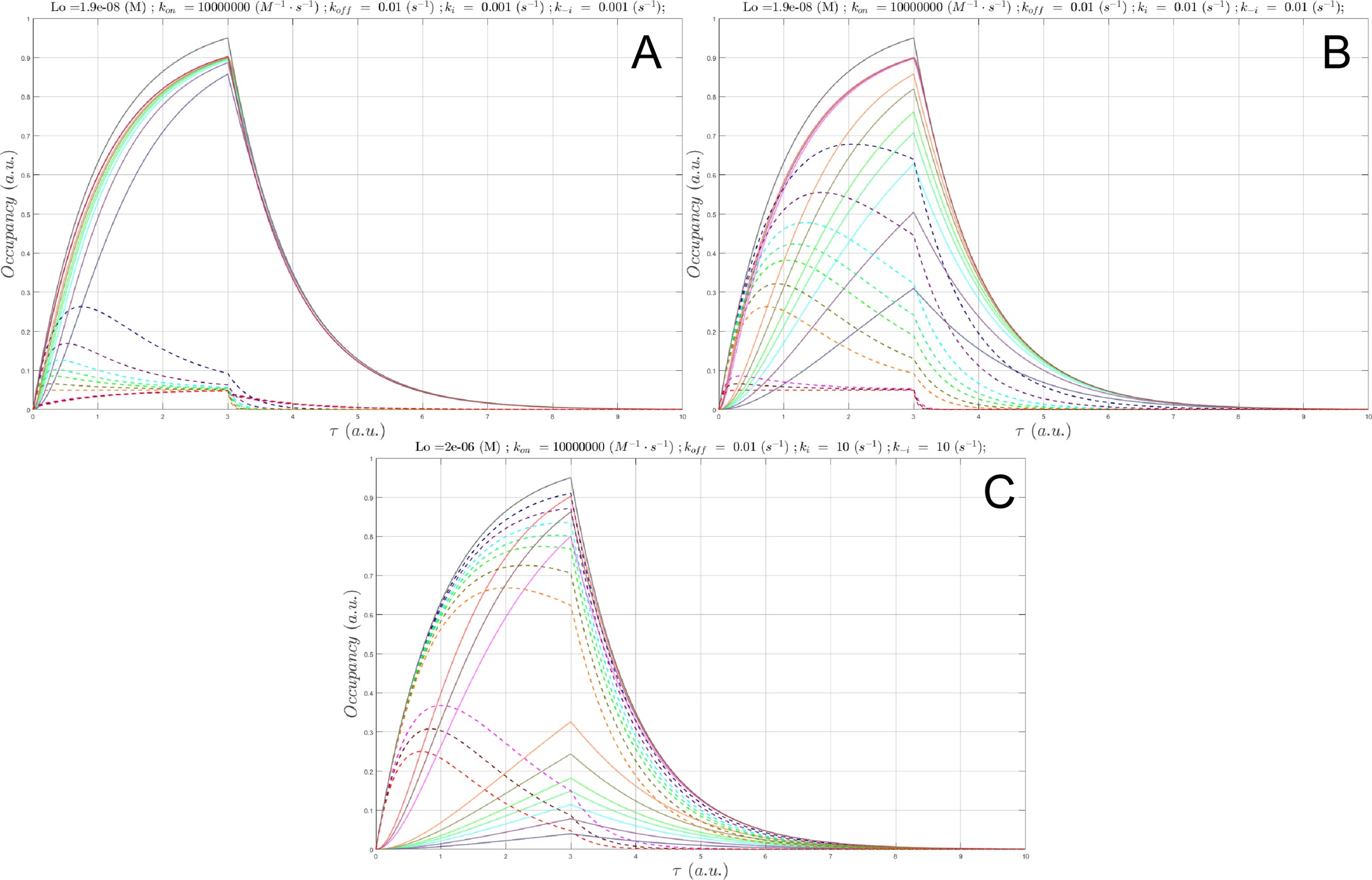

Supplement: S3 Fig — (a) Plot of Btotal(τ) (gray), c(τ) at a fixed L0 = 19 ⋅ Kd = 19 nM and Λ =50 min (ki = k−i = 10−3 s-1, konSS = 1x10^7^ M-1 s-1), in which kon was sampled between 1x10^5^ to 1x10^7^ M-1 s-1 (solid lines color-coded from blue to red according to increasing kon), and Bfree(τ) (dotted lines correspond to the c(τ) color scheme). (b) Same as (a), except Λ = 5 min (ki = k−i = 1x10-2 s-1, konSS = 1x10^8^ M-1 s-1). (c) Same as (a), except Λ = 300 ms (ki = k−i = 1x101 s-1, konSS = 1x10^11^ M-1 s-1) and L0 = 2 μM. Equilibrium occupancy (c95) is achieved at the fastest kon sampled (1x10^6^ to 1x10^7^ M-1 s-1), with the exception of Λ = 300 ms (ki = 1x101 s-1), which only reaches qSSO (even at L0 > 25 μM). (TIF) [file pone.0202376.s003.tif]

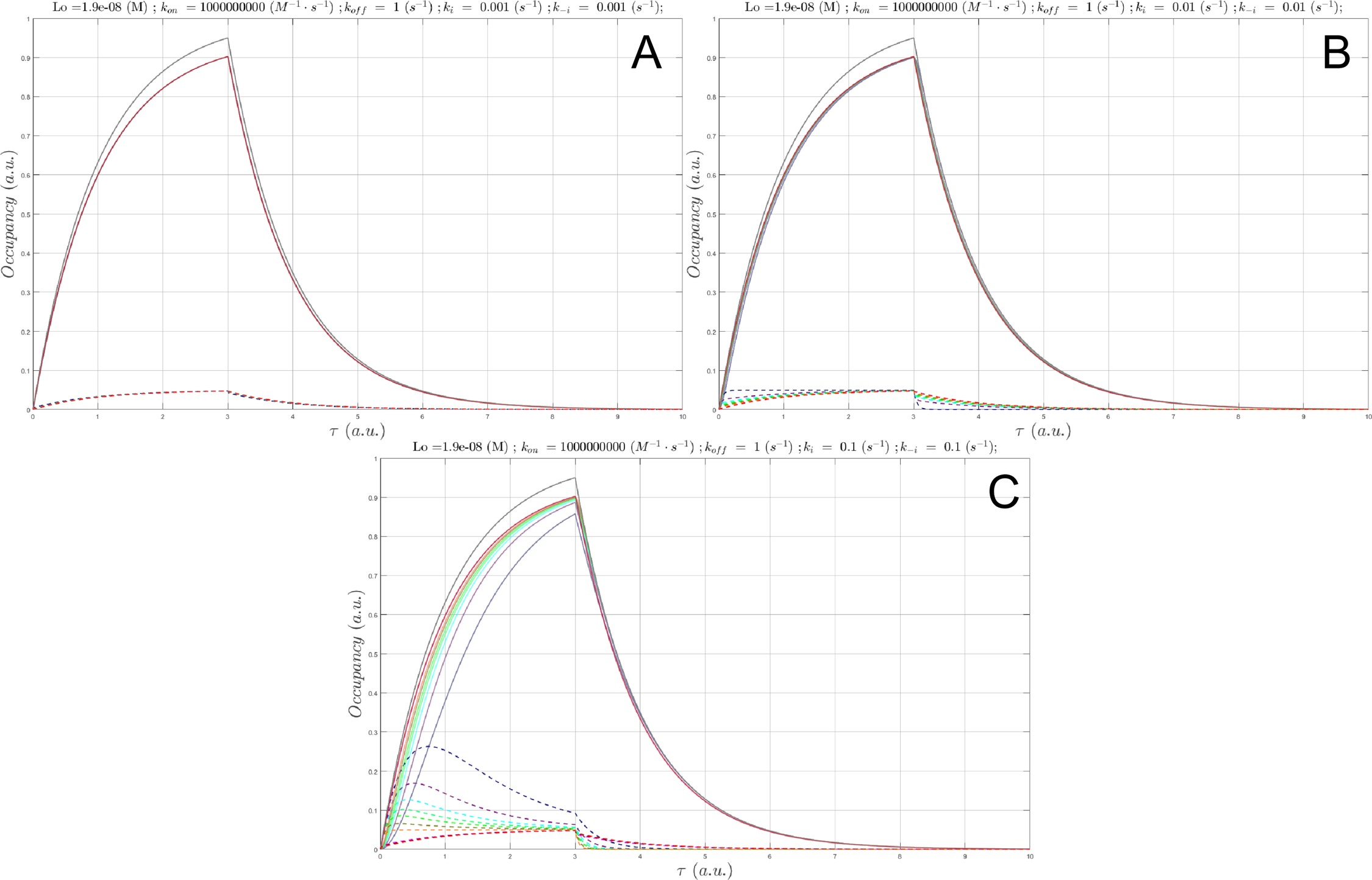

Supplement: S4 Fig — (a) Plot of Btotal(τ) (gray), c(τ) at a fixed L0 = 19 ⋅Kd = 19 nM and Λ = 50 min (ki = k−i = 1x10-3 s-1, konSS = 1x10^7^ M-1 s-1), in which kon was sampled between 1x10^7^ to 1x10^9^ M-1 s-1 (solid lines color-coded from blue to red according to increasing kon), and Bfree(τ) (dotted lines correspond to the c(τ) color scheme). b) Same as (a), except Λ = 5 min (ki = k−i = 1x10-2 s-1, konSS = 1x10^8^ M-1 s-1). (c) Same as (a), except Λ = 30 s (ki = k−i = 1x10-1 s-1, konSS = 1x10^9^ M-1 s-1). (TIF) [file pone.0202376.s004.tif]

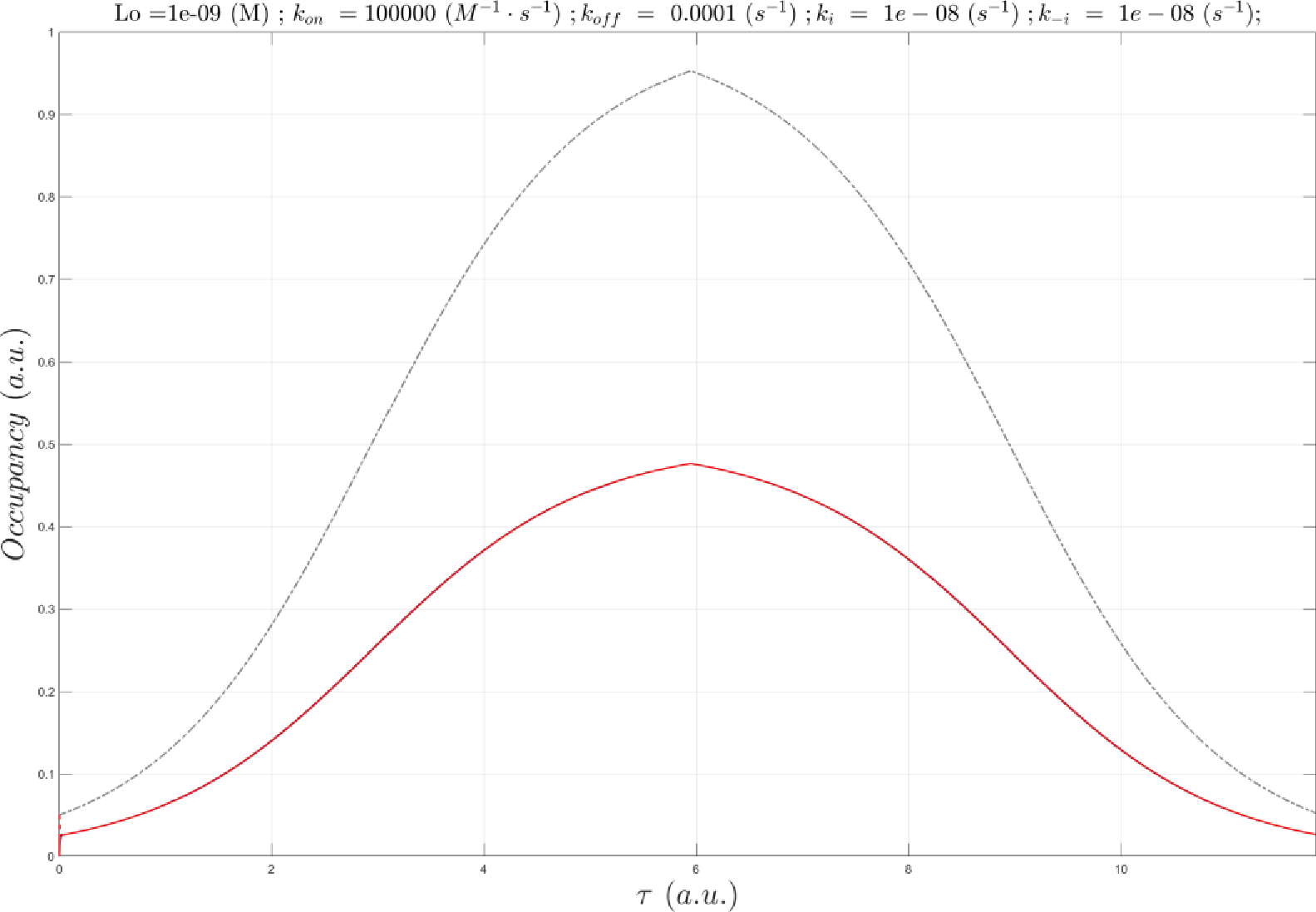

Supplement: S5 Fig — Plot of Btotal(τ) (gray), c(τ) at a fixed L0 = 1 nM and Λ = 38,508 hr (ki = k−i = 1x10-8 s-1), in which kon was sampled between 1x10^3^ M-1 s-1 to 1x10^5^ M-1 s-1 (solid lines color-coded from blue to red according to increasing kon), and Bfree(τ) (obscured by c(τ)). (TIF) [file pone.0202376.s005.tif]

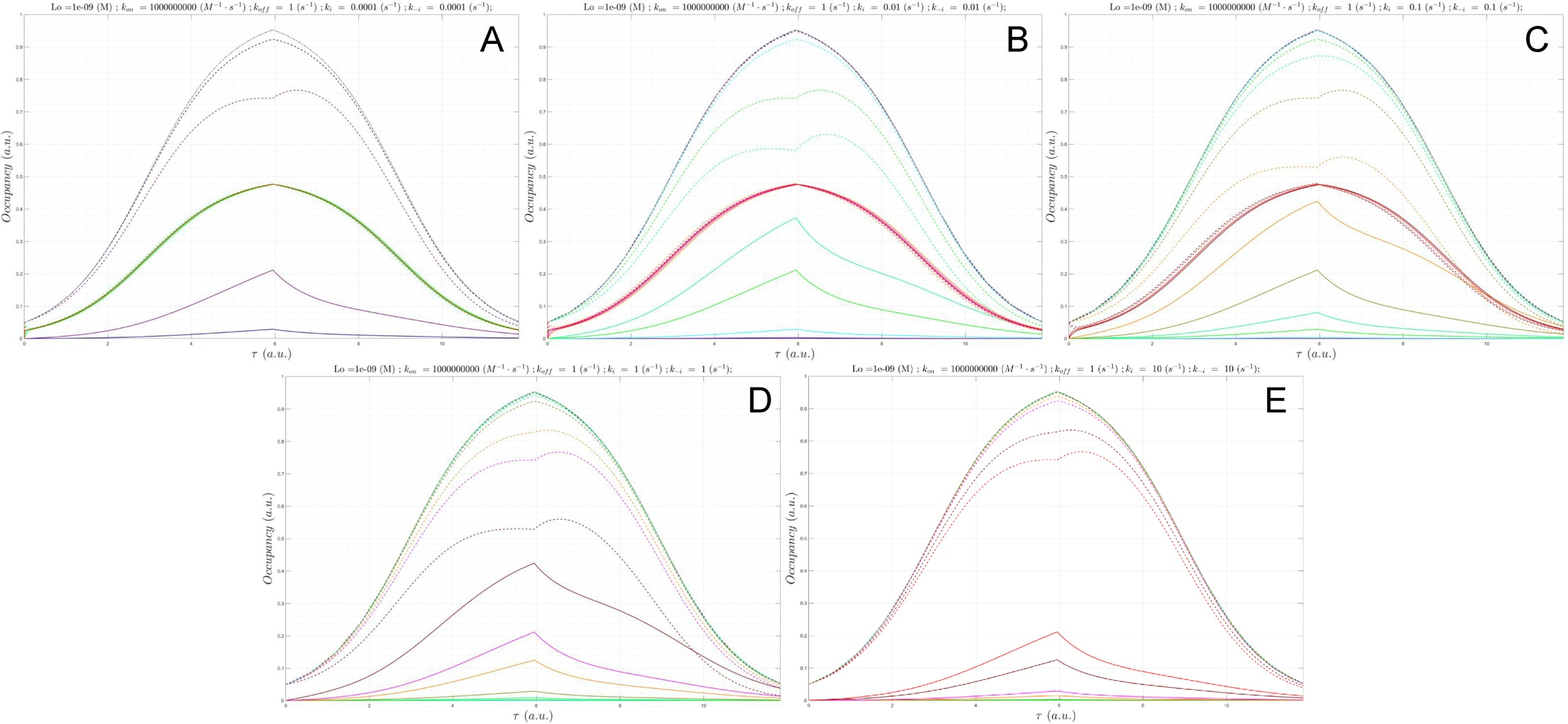

Supplement: S6 Fig — (a) Plot of Btotal(τ) (gray), c(τ) at a fixed L0 = 1 nM and Λ = 3.85 hr (ki = k−i = 1x10-4 s-1), in which kon was sampled between 1x10^4^ M-1 s-1 to 1x10^9^ M-1 s-1 (solid lines color-coded from blue to red according to increasing kon), and Bfree(τ) (dotted lines color coded the same as c(τ)). (b) Same as (a), except Λ = 2.3 min (ki = k−i = 1x10-2 s-1). (c) Same as (a), except Λ = 13.9 s (ki = k−i = 1x10-1 s-1). (d) Same as (a), except Λ = 1.39 s (ki = k−i = 1x100 s-1). (e) Same as (a), except Λ = 139 ms (ki = k−i = 1x101 s-1). (TIF) [file pone.0202376.s006.tif]

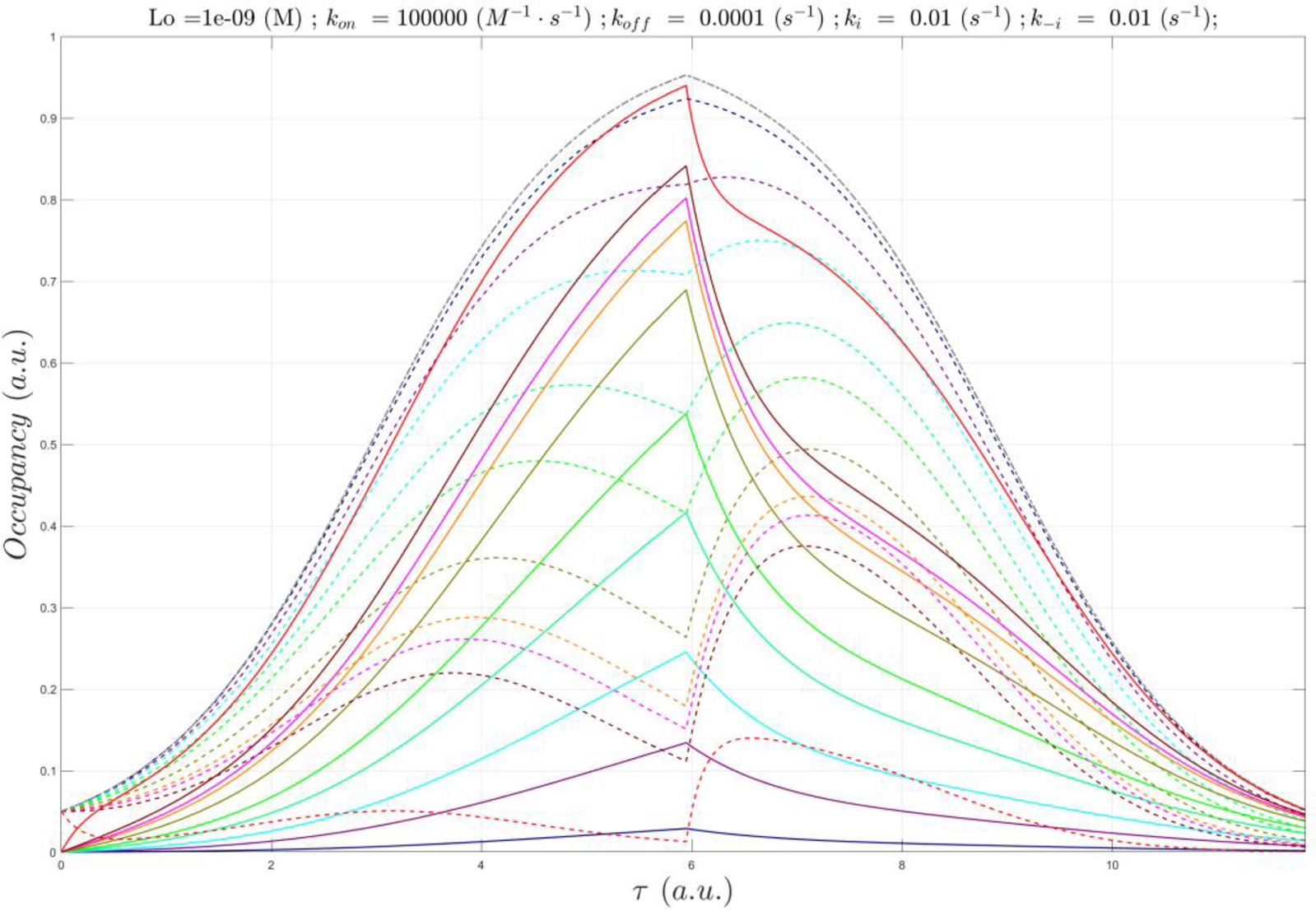

Supplement: S7 Fig — Plot of Btotal(τ) (gray), c(τ) at a fixed Kd = 1 nM, Λ = 2.3 min (ki = k−i = 1x10-2 s-1), koff = 1x10-5 s-1, and kon = 1x10^4^ M-1 s-1, in which L0 was sampled between 1 nM and 500 nM (solid lines), and Bfree(τ) (dotted lines color coded the same as c(τ)). kon < konSS is only compensated at sufficiently high L0, which in this case, qSSO at L0 = 500 nM. (TIF) [file pone.0202376.s007.tif]
